# Modulating the Activity of vmPFC Regulates Informational Social Conformity: A tDCS Study

**DOI:** 10.3389/fpsyg.2020.566977

**Published:** 2020-09-17

**Authors:** Yuzhen Li, Jinjin Wang, Hang Ye, Jun Luo

**Affiliations:** ^1^School of Economics, Zhejiang University of Finance and Economics, Hangzhou, China; ^2^Center for Economic Behavior and Decision-Making (CEBD), Zhejiang University of Finance and Economics, Hangzhou, China; ^3^School of Economics, Zhejiang University, Hangzhou, China; ^4^Interdisciplinary Center for Social Sciences (ICSS), Zhejiang University, Hangzhou, China

**Keywords:** social conformity, ventromedial prefrontal cortex, transcranial direct current stimulation, perceptual decision-making, informational influence

## Abstract

Social conformity has been evaluated in many different contexts, ranging from an emotional contagion in psychology, to speculative episodes in economics, to mass protests concerning politics. Previous neuroscience studies suggest that the ventromedial prefrontal cortex (vmPFC) participates in social conformity, especially when it comes to the value integration process, but the specific mechanism of vmPFC is still unclear. In this study, we aimed to identify a direct link between the vmPFC and conformity tendencies by means of transcranial direct current stimulation (tDCS). Conformity tendencies are measured by the probability that participants change their decisions when they observe the majority responses. In our experiment, subjects could make two decisions in each trial, once without social information and once with social information, which allowed us to directly observe the conformity tendency of subjects in different conditions. We found that cathodal stimulation of the vmPFC significantly increased conformity tendency and decreased response time when the initial decision of participants differs from the majority opinion. Based on the experimental results, our study suggests that the vmPFC mainly inhibits and regulates the informational conformity behavior. These findings complement investigations of the neural mechanism of conformity and the role of the vmPFC in the neural circuit behind conformity behavior.

## Introduction

Conformity is an influential and well-documented feature of human behavior in a number of domains, ranging from stock market bubbles and financial speculation to zealotry, political choice, and consumer preferences. According to social influence theory, human belief preferences and behaviors can be affected by observing the actions or outcomes of others (Lorenz et al., 2011; Muchnik et al., 2013). Conformity is a kind of social influence, and it refers to the act of changing one’s behavior to match the responses of others (Cialdini and Goldstein, 2004). There are many reasons for this behavior. People may follow the crowd because they think that the rest of the crowd is better informed, or they believe following others is more “safe” or “normal.” The behavior of conformity caused by the desire to form an accurate interpretation of reality and to behave correctly is called informational social conformity, which is the focus of our research.

Uncertainty is inherent in all biological systems (Dall et al., 2005; Bach and Dolan, 2012). Individuals usually need to integrate information in uncertain environments to make accurate decisions based on the current environmental states (Toelch and Dolan, 2015). Both private and social information may be incomplete and imperfect. In a complete information environment, a rational decision maker can act strictly in accordance with the Bayesian principle, but due to objective conditions, we cannot be sure of the uncertainty and reliability of all information sources (De Martino et al., 2017; Mahmoodi et al., 2018). In such situations, an individual’s subjective belief in various sources of information before making a decision may directly affect the decision-making outcome. In real life, we can also easily find that subjective confidence has a great effect on the extent to which we will be influenced by others, especially when the opinions of others conflict with our own (De Martino et al., 2013; Boldt et al., 2019). Therefore, a possible reasonable conjecture is that the degree of individual subjective confidence has a negative effect on its conformity tendency, that is, the lack of self-confidence may lead to an increase in conformity tendency, and vice versa. However, it is still unclear what role confidence plays in conformity behavior.

Since many of our judgments are based on subjective perception and value estimation, there will inevitably be some deviations from normative assumptions. Previous research has suggested that rational choice models are often not descriptive of human behavior (Simon, 1996; Elqayam and Evans, 2011; Brighton and Gigerenzer, 2012). In particular, when facing multiple sources of information, participants assign overproportionally weight to private information depending on their own accuracy (Huck and Oechssler, 2000; Toelch et al., 2014; Niu et al., 2019), individual predisposition and other factors (Huber et al., 2014). The phenomenon that the behavior of the subjects systematically deviates from the Bayesian Nash Equilibrium (BNE) attracted the attention of researchers (Kübler and Weizsäcker, 2004; Walden and Browne, 2009; Weizsäcker, 2010). Therefore, investigating neural mechanisms to improve our understanding of value estimation in information integration is useful and can provide important, general insights into the study of social decision-making.

Asch (1956) conducted a perceptual experiment on conformity, and this classic methodology was subsequently adopted by many researchers. Berns et al. (2005) used a mental rotation task to investigate the neural basis of individualistic and conforming behavior in the face of wrong information. Toelch et al. (2014) applied a perceptual task that required players to guess the location of a briefly flashed stimulus to identify the neural substrate of an optimal exploitation of social information under uncertainty. Germar et al. (2014) employed a perceptual decision-making task to identify whether a visual stimulus was dominated by the color orange or blue to analyze the specific cognitive mechanisms mediating changes in individuals’ opinions. Motivated by the above literature regarding social influence, cognitive mechanisms and neuronal substrates, we extend this perceptual experiment design in which subjects need to make the right decision as often as possible after receiving individual and externally generated (i.e., social) information. In each trial of our experiment, subjects first needed to independently make a visual recognition response and then they could make a decision again after being informed of others’ responses. By collecting the data on the changes in the subjects’ beliefs, we could then directly analyze the conformity tendency of the subjects after they knew the majority responses under different conditions.

To date, many studies have begun to shed light on the neural mechanisms underlying social conformity, most of which are based on functional magnetic resonance imaging (Schnuerch and Gibbons, 2014). The ventral striatum and ventromedial prefrontal cortex (vmPFC) have exhibited activation when participants learned about a majority opinion or want to be in agreement with them (Cooper et al., 2013). Studies on misalignment (Bahrami et al., 2010; Mahmoodi et al., 2015) show that deviation from social group norms often evokes activity in the dorsal medial prefrontal cortex (dmPFC) and dorsal anterior cingulate cortex (dACC). Neuroimaging shows increasing activity in the anterior insula during violations of expectation in both social and nonsocial contexts (Chang and Sanfey, 2011; Lieberman and Eisenberger, 2015). Berns et al. (2010) reported that those who have a stronger conformity tendency showed higher activation in the posterior medial frontal cortex (pMFC) and insula when other people’s opinions were shown, regardless of the degree of the mismatch. Ruff and Fehr (2014) suggested that social valuation computations in the vmPFC may depend on input from specialized regions, as the orbitofrontal cortex (OFC) showed functional connectivity with the anterior insula during voluntary giving decisions. In addition, previous studies have found that vmPFC is related to value estimation and value calculation (Smith et al., 2010; Levy and Glimcher, 2012; Clithero and Rangel, 2014), especially when value comparison and confidence factors are involved in the value estimation process (Rolls et al., 2010; De Martino et al., 2013; Donoso et al., 2014; Lebreton et al., 2015). Thus the vmPFC seems to mediate informational conformity behavior, although a better understanding will need further causal relationship investigations.

Overall, the current study aimed to investigate the effect of vmPFC activity on informational social conformity from the perspective of neuroscience. To formally test the causal relationship, we conducted a perceptual experiment to study whether and how transcranial direct current stimulation (tDCS) over the vmPFC affected informational social conformity. In addition, we compared the actual conformity tendency of the subjects with the Bayesian estimation model to study individuals’ integration between private and social information.

## Materials and Methods

### Participants

Sixty-four subjects were recruited. Three subjects were excluded because they did not complete all experimental tasks. In the end, we collected data from 61 participants (31 females, average age = 20.18 years). The experiment lasted approximately 1 h, and each participant received an average payment of 50 RMB yuan (approximately 7.06 United States dollars) after the experiment. All of the participants were right-handed, had no history of psychiatric illness or psychiatric problems, had normal or corrected-to-normal vision and were naïve to tDCS and our decision-making task. No participants reported any adverse side effects regarding pain in the scalp or headaches after the experiment. The study was approved by the Ethics Committee of Zhejiang University of Finance and Economics.

### Experimental Tasks and Procedure

**FIGURE 1:**
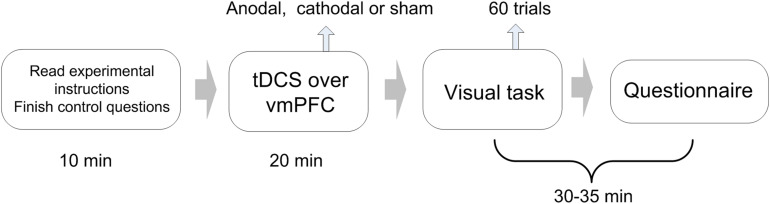
Experimental design.

**FIGURE 2:**
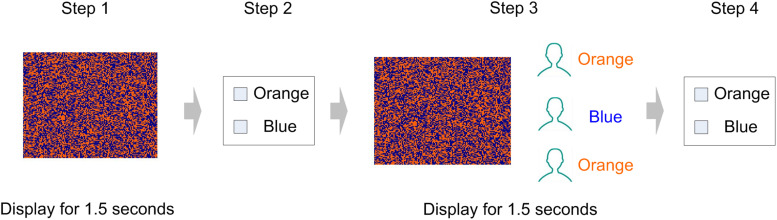
Visual task (1 trial). In the example shown, the picture is dominated by blue blocks. In step 2 and step 4, the participant responded with a button press, indicating whether the picture is dominated by blue or orange. In step 3, two participants in the group have reported that the image was dominated by orange, and one participant has reported that the image was dominated by blue.

Participants were asked to complete an experiment related to visual recognition. The experiment consisted of 60 visual task trials (Figure 1). Our visual task was adapted from the perceptual decision-making experiment designed by Germar et al. (2014), to which we made some changes according to the purpose of the present experiment (Figure 2). Specifically, participants were asked to determine whether an image on the screen has more blue blocks or more orange blocks. Each image consists of 128 × 128 color blocks.

Subjects were asked to take seats randomly. During the session, they were separated by partitions, and communication with each other was prohibited. Every four subjects in the laboratory were randomly divided into groups, and the team members would not change during the entire experiment. The experiment included 60 trials of visual tasks. The complete process of each trial is as follows (Figure 2):

Step 1: All participants observe a picture on the screen (displayed for 1.5 s).
Step 2: The picture disappears, and participants have to decide whether the picture was dominated by the color orange or blue (no time limit).
Step 3: Four participants in the same group observe the picture presented in the first step again and are informed of the others’ decisions in step 1 (displayed for 1.5 s).
Step 4: The picture and the others’ decisions disappear, and participants make decisions for the second time (no time limit).

Participants were told that their individual computers were connected via a server, which will collect and display participants’ decisions. In reality, all participants completed the task individually on stand-alone computers. The displayed responses on each trial were not actual responses of the other participants but were generated by the experimental software (Germar et al., 2014). We created a design matrix (2 × 2 × 2 = 8 trials; Table 1) by using all combinations of conditions (incongruent vs. congruent), absolute net public information (the difference between the number of orange choices and blue choices; 3 vs. 1) and correctness of majority response (right vs. wrong).

**TABLE 1:** Main experimental design.

Condition	Net public information	Majority response
Incongruent	NPI = −3	Right/wrong
	NPI = −1	Right/wrong

Congruent	NPI = 3	Right/wrong
	NPI = 1	Right/wrong

Following the design from Huber et al. (2014), we manipulated subjects’ coherent environment in two conditions (congruent vs. incongruent) so that the subject’s first decision was congruent with the majority in some trials and incongruent in the other trials. We also adopted a variable called Net Public Information (NPI) designed by Frydman and Krajbich (2017). NPI >0 indicates that the participant received a congruent majority response (the participant’s decision was the same as that of most people in the group), while NPI <0 indicates an incongruent majority response. The NPI had four distinct values in our study, which were 1 (two in the group provided the same response as the participant, one responded differently) and 3 (all three others in the group provided the same response as the participant) in the congruent condition; −1 (two in the group provided a different response than the participant, one provided the same response) and −3 (all three others in the group provided a different response than the participant) in the incongruent condition. In particular, for all 60 trials, all of the other three participants’ responses were evenly distributed among all right (for 15 trials); two were right and one was wrong (for 15 trials), two were wrong and one was right (for 15 trials); all three were wrong (for 15 trials). Half of the stimulus pictures were dominated by blue blocks, and the other half of the stimulus pictures were dominated by orange blocks. The dominant color of the picture and the different responses of other participants were ordered randomly.

There was no time limit for the decision phase, but participants were encouraged to complete it as soon as possible. To motivate participants to seriously implement every decision in the experiment, the system randomly selected one of the two decisions (the first decision or the second decision) in each trial of experiments to check whether the decision was correct. If the decision was correct, the participant was considered successful in this trial of the experiment and would benefit from it (1 RMB yuan per trial).

### tDCS

tDCS is a non-invasive form of neuromodulation that has been demonstrated to modulate a variety of cognitive functions by changing cortical excitability (Kuo et al., 2014; Lefaucheur et al., 2017). In general, anodal stimulation enhances cortical excitability, whereas cathodal stimulation reduces cortical excitability (Nitsche and Paulus, 2000). tDCS applies a very weak direct current via two saline-soaked surface sponge electrodes (5 cm × 7 cm; 35 cm^2^) to the scalp, modulating the cortical excitability and therefore influencing subjects’ brain functions. Specifically, we used a tDCS device (NeuroConn, Ilmenau, Germany) to modulate the subjects’ cortical excitability of the vmPFC.

**FIGURE 3:**
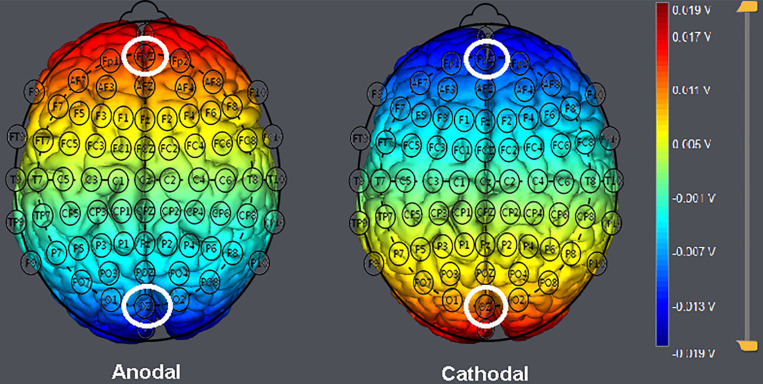
Locations of the electrodes and stimulation modes in transcranial direct current stimulation (tDCS) treatments. Schematic of electrode positions Fpz and Oz based on the international electroencephalography (EEG) 10–20 system of the human brain. The shading represents the range of input voltage from −0.019 to 0.019 V.

Participants were randomly assigned to one of the three stimulation treatments. In line with previous neuroscience research targeting the vmPFC (Zheng et al., 2016; Gilam et al., 2018; Adelhöfer and Beste, 2020), the anodal electrode was placed over the Fpz position according to the international 10–20 system for electrode placement, while the cathodal return electrode was placed over the Oz position (Sellaro et al., 2015) in the anodal stimulation group (*n* = 20, 10 males and 10 females). For cathodal stimulation (*n* = 20, 10 males and 10 females), the polarity was reversed (Figure 3). The stimulation lasted for 20 min. The current was constant and had an intensity of 1.5 mA intensity with 30 s of ramp up and down. The safety and effectiveness of these parameter settings have been shown in previous studies (Nitsche et al., 2003, 2008; Sellaro et al., 2016). For sham stimulation (*n* = 21, 10 males and 11 females), the procedures were the same, but the current lasted only for the first 30 s. There was actually no current for the rest of the stimulation period, and subjects were unaware of it. This method of sham stimulation has been shown to be reliable (Gandiga et al., 2006). After the stimulation, the tDCS device was taken off, and the participant was asked to complete several perception tasks.

## Computational Model

Our decision-making computational model borrows several key constructs from Bayesian perceptual and value-based decision-making. Bayesian explanations have long been an important approach for studying belief updates and information integration and provide normative principles for the above behaviors. The fundamental concept behind the Bayesian approach is to interpret the current evidence in light of all available prior knowledge using the rules of probability (Kriegeskorte and Douglas, 2018).

For our analysis, we needed a concrete model that would allow participants to integrate information efficiently from different sensory cues and to propagate information from one stage of processing to another. In particular, a Bayesian decision-maker would integrate the information from several unbiased individuals, giving equal weight to each decision, assuming that all participants have similar correct rates (Toelch and Dolan, 2015). Furthermore, given the responses of other participants, the posterior probability that following the majority is correct (Bayesian Nash Equilibrium, BNE) would be:

(1) $$p\,\left(c\,o\,r\,r\,e\,c\,t|n,m\right)=\frac{p_{c}^{m}*{\left(1-p_{c}\right)}^{n}}{p_{c}^{m}*{\left(1-p_{c}\right)}^{n}+{\left(1-p_{c}\right)}^{m}*p_{c}^{n}}$$

where *p*_*c*_ is the perceived correct rate (Toelch and Dolan, 2015). To explore the weight distribution of individuals among different information sources, here we mainly focus on the information (private and social) conflict situation (incongruent condition). When NPI <0 (under incongruent condition), n is the number of participants who made the same decision in step 1 as the individual (including her/himself), and m is the number of participants who made the other decision.

However, when dealing with problems in practice, people may not assign the same weight to each information source as in Equation (1), especially when faced with different majority opinions. Therefore, we refer to the information heteroweighting model of Huber et al. (2014) and Niu et al. (2019) to set the weight given to the private information as β (instead of 1); then, under incongruent conditions, the posterior probability in Equation (1) is:

(2) $$p_{\beta }\,\left(c\,o\,r\,r\,e\,c\,t|n,m\right)=\frac{p_{c}^{m}*{\left(1-p_{c}\right)}^{\beta }*{\left(1-p_{c}\right)}^{n-1}}{p_{c}^{m}*{\left(1-p_{c}\right)}^{\beta }*{\left(1-p_{c}\right)}^{n-1}+p_{c}^{\beta }*p_{c}^{n-1}*{\left(1-p_{c}\right)}^{m}}$$

When 1 > *p*_*c*_ > 0.5,

$$\frac{d\,p_{\beta }\,\left(c\,o\,r\,r\,e\,c\,t|n,m\right)}{d\,\beta }\;=\frac{\left[\ln\left(1-p_{c}\right)-\ln p_{c}\right]*{\left(1-p_{c}\right)}^{\beta +m+n-1}*p_{c}^{\beta +m+n-1}}{{\left[p_{c}^{m}*{\left(1-p_{c}\right)}^{\beta +n-1}+p_{c}^{\beta +n-1}*{\left(1-p_{c}\right)}^{m}\right]}^{2}}<0.$$

Where $\frac{d\,p_{\beta }\,\left(c\,o\,r\,r\,e\,c\,t|n,m\right)}{d\,\beta }$ is the derivative of *p*_β_(*correct*|*n*,*m*) with respect to β. Therefore, when the individual assigns higher weight to private information, then β > 1, and the conformity tendency is lower than BNE (Equation 1); when subjects rely more on social information, then β < 1, and the conformity tendency is higher than BNE.

## Data Analysis

The critical variables are whether the participants changed their decisions after learning about the responses of others, which reflect the subjects’ conformity tendency under different stimulations (anodal vs. sham vs. cathodal), and mean response time (RT). Condition (incongruent vs. congruent), absolute NPI (3 vs. 1) and correctness of majority response (right vs. wrong) were within-subject factors, and stimulation (anodal vs. sham vs. cathodal) was a between-subject factor. Choice was coded as a dummy variable and was set to 1 if a participant made a conformity choice and 0 otherwise. We will analyze the conformity tendencies under the influence of the above factors. The different trials and all possible responses in the experiment are shown in Table 2. The percentages of responses consistent with the majority were not normally distributed, as assessed by Shapiro-Wilk’s test (*p* < 0.05). Thus, we performed non-parametric tests to analyze the data. The data were statistically evaluated using SPSS software (version 22) and Stata statistical software (version 14.0).

**TABLE 2:** Participant decision table.

Congruency	NPI	First response	Majority response	Second response	Response shift	Conformity
Incongruent	NPI = −3	Wrong	Right	Right	Shift	Conformity
	NPI = −1					
	NPI = −3	Right	Wrong	Wrong		
	NPI = −1					
Congruent	NPI = 3	Right	Right	Right	No shift	
	NPI = 1					
	NPI = 3	Wrong	Wrong	Wrong		
	NPI = 1					

Incongruent	NPI = −3	Right	Wrong	Right	No shift	No conformity
	NPI = −1					
	NPI = −3	Wrong	Right	Wrong		
	NPI = −1					
Congruent	NPI = 3	Right	Right	Wrong	Shift	
	NPI = 1					
	NPI = 3	Wrong	Wrong	Right		
	NPI = 1					

## Results

### Conformity Tendency Analysis: Baseline (Sham Group) Results

In the sham stimulation group, there was a phenomenon in which the subjects changed their decisions by following the social information (*p* < 0.001). The percentage of responses consistent with the majority was strongly influenced by the congruency between the initial independent decision and the majority opinion of others in the group (social information). The Wilcoxon signed-rank test suggested that participants were more likely to follow social information under congruent conditions than under incongruent conditions (congruent vs. incongruent: *p* < 0.001; Table 3). The tendency for a participant to make a decision consistent with the majority also held for trials in the incongruent condition [χ^2^(3) = 71.052, *p* < 0.001; NPI = −3 vs. NPI = −1: FDR-adjusted *p* = 0.007].

**TABLE 3:** Participants’ conformity tendency in different conditions.

	M	(SE)	Bayes’ rule (based on the estimated correct rate)		M	(SE)
NPI < 0	0.356	(0.036)	–	NPI > 0	0.985	(0.007)
NPI = −3	0.562	(0.053)	0.604	NPI = 3	1.000	(0.000)
NPI = −1	0.138	(0.035)	0.500	NPI = 1	0.973	(0.012)

Participants were asked to give an estimate of the accuracy of their first responses in the post-experiment questionnaire. We found that participants’ estimation of their correct rate is significantly lower than the actual correct rate (average actual correct rate vs. estimated correct rate: 80.40 vs. 55.24%; *p* < 0.001). According to the estimated correct rate of first responses (55.24%), we can calculate the posterior probabilities when following the majority is the correct strategy according to Bayes’ rule (Equation 1). The comparison of the BNE and the actual probability of the subjects’ conformity behavior (see Table 3) indicated that participants deviated from the Bayesian model. The experimental data show that the conformity tendencies are usually below the Bayesian estimates under incongruent conditions especially when NPI = −1.

### Conformity Tendency Analysis: tDCS Stimulation Results

**FIGURE 4:**
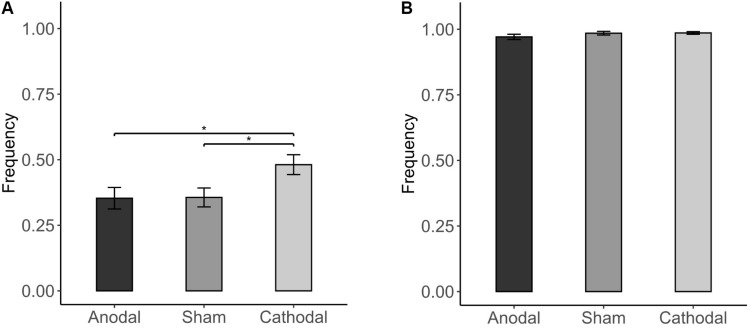
Impact of stimulation on conformity tendency. **(A)** Incongruent condition: Cathodal stimulation led to a higher percentage of responses consistent with the majority than both sham and anodal stimulation in the incongruent condition. **(B)** Congruent condition: There was no significant difference among the stimulation in the congruent condition. Error bars indicate ± 1 SEM. Asterisks indicate statistically significant differences between the treatments. *P < 0.05; **P < 0.01; ***P < 0.001.

The results showed that conformity tendencies from all three stimulation conditions were significantly different from zero (anodal: *p* < 0.001; cathodal: *p* < 0.001; sham: *p* < 0.001). Participants’ conformity tendency was affected by stimulation of the vmPFC [χ^2^(2) = 7.600, *p* = 0.022; Figure 4]. For the incongruent condition, cathodal vmPFC stimulation resulted in a significantly higher percentage of responses in line with the majority than both sham stimulation (48.053 vs. 35.643%; FDR-adjusted *p* = 0.016) and anodal stimulation (48.053 vs. 35.283%; FDR-adjusted *p* = 0.016), indicating that cathodal tDCS stimulation increased conformity tendencies (Figure 4A). For the congruent condition, almost all participants chose to be consistent with the majority (Figure 4B). The results also confirmed that the conformity tendency in response to cathodal stimulation was significantly higher than that in response to both sham stimulation and anodal stimulation when NPI = −3 [χ^2^(2) = 7.402, *p* = 0.025; Table 4].

**TABLE 4:** Effect of tDCS on the conformity tendency in different NPIs.

	1 Anodal		2 Sham		3 Cathodal		
Stimulation	M	(SE)	M	(SE)	M	(SE)	*Post-hoc* tests
NPI = −3	0.511	(0.054)	0.562	(0.053)	0.711	(0.046)	1 and 2, 1–3* and 2 and 3*
NPI = −1	0.184	(0.041)	0.138	(0.035)	0.223	(0.046)	1 and 2, 1–3 and 2 and 3
NPI = 3	0.986	(0.008)	1.000	(0.000)	0.996	(0.004)	1 and 2, 1–3 and 2 and 3
NPI = 1	0.957	(0.015)	0.973	(0.012)	0.977	(0.009)	1 and 2, 1–3 and 2 and 3

Asterisks indicate statistically significant differences between the treatments. *P < 0.05; **P < 0.01; ***P < 0.001.

The conformity tendency of participants held for trials in the incongruent condition [NPI = −3 vs. NPI = −1: FDR-adjusted *p* < 0.001; χ^2^(3) = 199.376, *p* < 0.001]. Participants were more likely to make a decision in line with the majority, as absolute NPI increased in the incongruent condition (NPI = −3, 59.464%; NPI = −1, 18.189%). In addition, participants were more likely to follow the right majority responses than the wrong majority responses (61.852 vs. 34.212%, *p* < 0.001).

**FIGURE 5:**
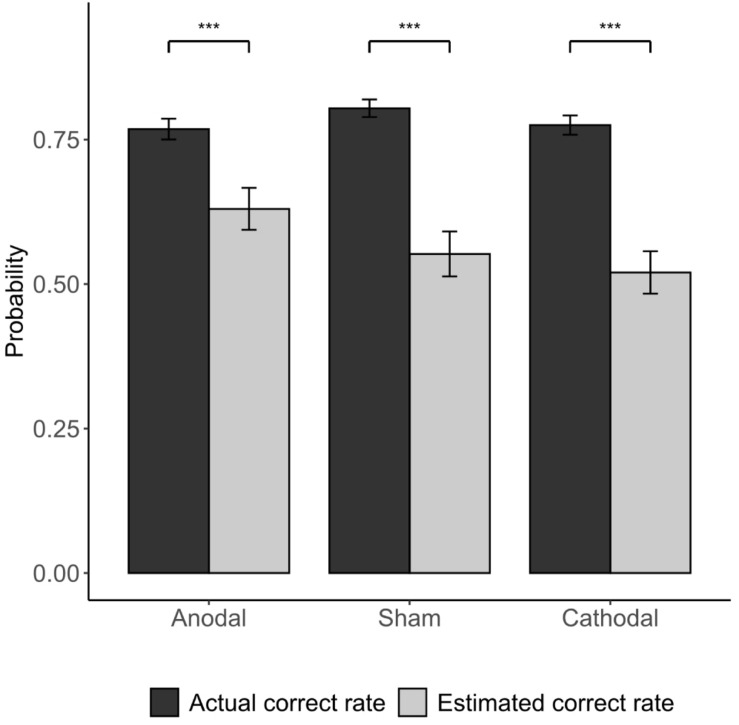
Actual correct rate vs. estimated correct rate of participants. Error bars indicate ± 1 SEM. Asterisks indicate statistically significant differences between the actual and estimated correct rate. *P < 0.05; **P < 0.01; ***P < 0.001.

**FIGURE 6:**
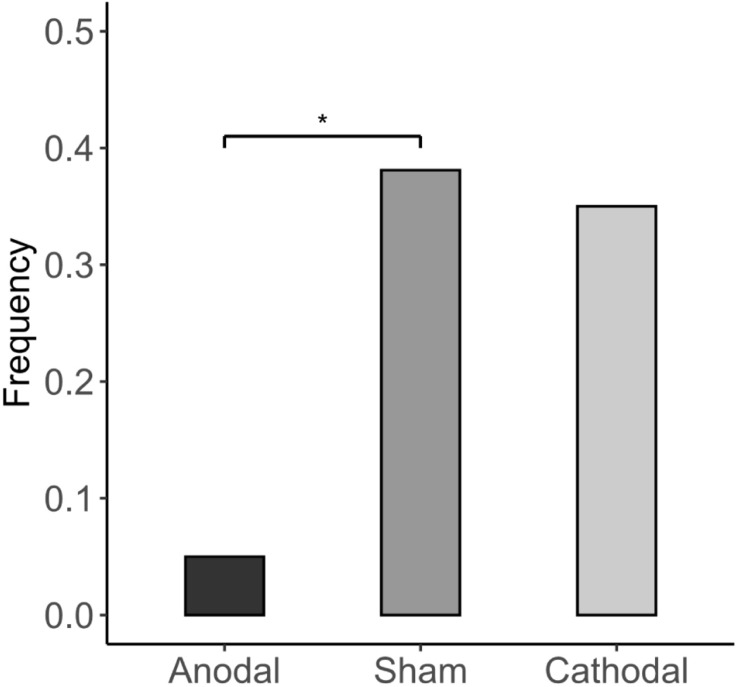
Estimates of others’ performance. The proportion of subjects who think they have a lower correct rate than others for the first response. Asterisks indicate statistically significant differences between the treatments. *P < 0.05; **P < 0.01; ***P < 0.001.

The Kruskal-Wallis H test revealed that different stimulations did not significantly affect the accuracy of the subjects’ first responses (anodal: correct rate = 0.768; sham: correct rate = 0.804; cathodal: correct rate = 0.775; *p* > 0.1). This indicates that the stimulation of the vmPFC did not change the subject’s ability to identify the dominant color in the experiment. Participants’ estimation of their correct rate is significantly lower than the actual correct rate in all three stimulation groups (Figure 5). Furthermore, we investigated how the subjects felt regarding whether the accuracy of their first responses was comparable to those of others. In the cathodal and sham stimulation groups, more than one-third of the participants thought that their correct rate was lower than most others, while in the anodal stimulation group, only 5% thought so [anodal, *p* = 5%; sham, *p* = 38.1%; cathodal, *p* = 35%; χ^2^(2) = 6.982, *p* = 0.03; anodal vs. sham: FDR-adjusted *p* = 0.027; Figure 6].

**FIGURE 7:**
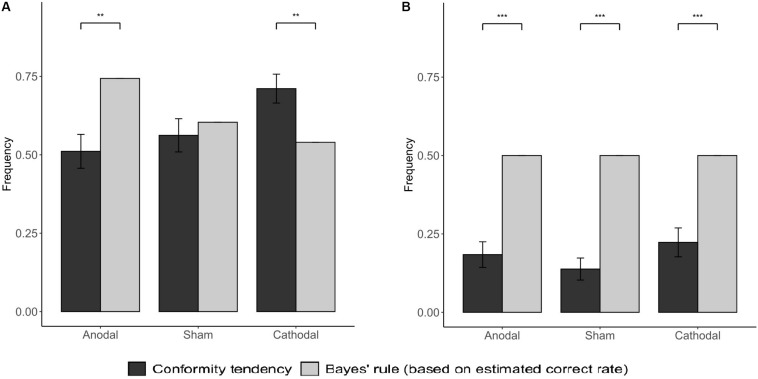
Social conformity tendency in subjects and from BNE. The gray bars indicate the Bayesian Nash Equilibrium (based on estimated correct rate). **(A)** was for NPI = −3; **(B)** was for NPI = −1. Error bars indicate ± 1 SEM. Asterisks indicate statistically significant differences between the BNE and the actual probability of the subjects’ conformity behavior. *P < 0.05; **P < 0.01; ***P < 0.001.

The results of Bayesian model analysis show that subjects in different stimulation groups have significantly different information weight distribution tendencies. When NPI = −3, the conformity tendency of the subjects under the anodal stimulation is significantly lower than the BNE (*p* = 0.002), indicating that the subjects assign a higher weight to the results of private judgment. Conversely, the subjects in the cathodal stimulation group had higher conformity tendency than BNE, which indicates that the subjects in this group give higher weight to social information (*p* = 0.005; Figure 7A). When NPI = −1, the conformity tendency of subjects in the three stimulation groups was significantly lower than that of BNE (anodal: *p* < 0.001; sham: *p* < 0.001; cathodal: *p* < 0.001; Figure 7B). Based on the above results, we suggest that the stimulation of vmPFC may change the subjects’ conformity tendency by changing the weight distribution between the subjects’ private and social information.

### Response Time Analysis: tDCS Stimulation Results

**FIGURE 8:**
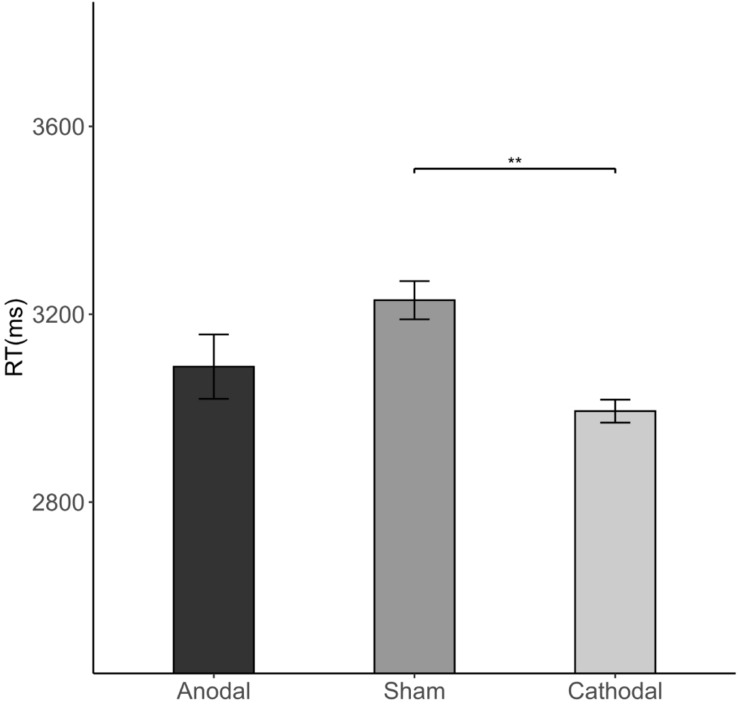
Impact of stimulation on the RT. Error bars indicate ± 1 SEM. Asterisks indicate statistically significant differences between the treatments. *P < 0.05; **P < 0.01; ***P < 0.001.

The Kruskal-Wallis H test revealed that there were differences in average RT (length of the decision time in step 4) among the three stimulations [χ^2^(2) = 8.733, *p* = 0.013; Figure 8]. Cathodal stimulation resulted in a shorter average RT than that of sham stimulation (2.994 vs. 3.23 s, FDR-adjusted *p* = 0.005).

**FIGURE 9:**
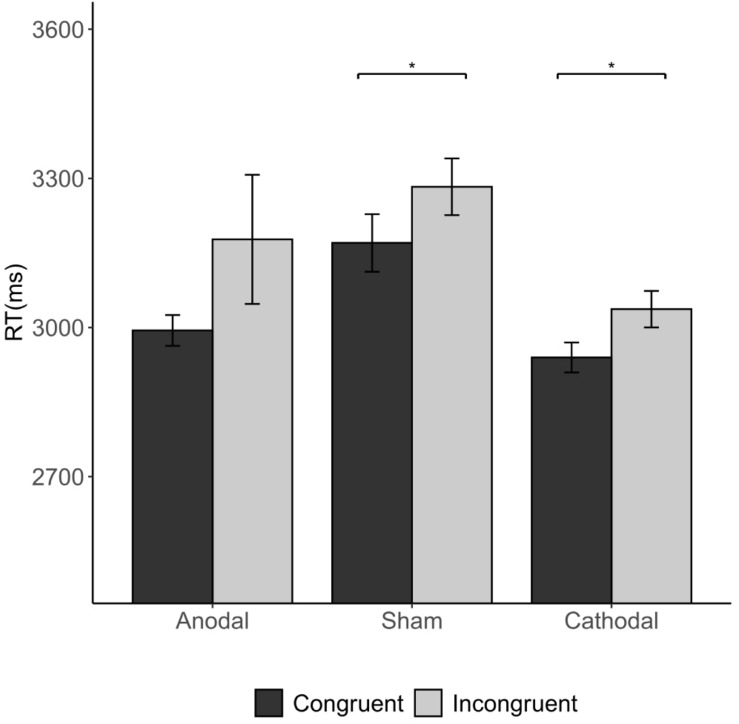
Impact of congruency condition and stimulation on the RT. Error bars indicate ± 1 SEM. Asterisks indicate statistically significant differences between the congruent and incongruent conditions. *P < 0.05; **P < 0.01; ***P < 0.001.

The Wilcoxon signed-rank tests showed that total average RTs differed significantly between congruent conditions and incongruent conditions (3.038 s vs. 3.165 s; congruent vs. incongruent condition *p* = 0.033). This difference was significant in both the sham stimulation (*p* = 0.028) and the cathodal stimulation group (*p* = 0.042), indicating that RTs were overall longer in incongruent conditions than in congruent conditions (Figure 9). This indicates that when the subjects were informed of a majority of different opinions, the RT was longer than that of a majority of the same opinion.

## Discussion

Research on the neural mechanism of conformity behavior can help us to understand the roles played by social-psychological factors in many important social behaviors. Previous research generally believed that conformity is the consequence of an informational or normative influence (Deutsch and Gerard, 1955). An informational influence (based on factual information) should predominate when the issue is intellective (concerned with achieving a correct answer), while a normative influence should predominate when the issue is judgmental (concerned with making “preferred” or “proper” choices). However, recent research findings on the two types of conformity are interrelated and often difficult to disentangle theoretically as well as empirically (Cialdini and Goldstein, 2004; Toelch and Dolan, 2015). In the present experiment, we employed tDCS over the vmPFC to assess the role of the stimulated brain area in an individual’s informational conformity tendency under different conditions during a perceptual task.

The vmPFC has been shown to integrate signals from different sources and to encode the value of a chosen option during decision-making (Tom et al., 2007; Rangel and Hare, 2010; Padoaschioppa, 2011; Rushworth et al., 2011). Hunt et al. (2012) found that the vmPFC was selectively activated in trials in which subjects had to combine probability and magnitude information to choose accurately. Clithero and Rangel (2014) demonstrate the centrality of the vmPFC in the computation of values across tasks, reward modalities and stages of the decision-making process. Ruff and Fehr (2014) suggest that vmPFC activity reflects valuation during decision making. In particular, vmPFC is related to the computations of the signals of all anticipated values and costs associated with the different options that are then integrated into a single quantity that is utilized to make the choice. Ting et al. (2015) suggest that an important function of the mPFC is to support social inference and prospective thinking by encoding the probability of future events. In addition, vmPFC has also been found to be related to subjective confidence during the value integration process. De Martino et al. (2013) found that vmPFC activities are related to value comparison and confidence in the value-based decision-making process. Lebreton et al. (2015) demonstrate that the activities in vmPFC are not only related to subjective values in overt judgments, but also participate in coding confidence.

In this article, we examined the contribution of the vmPFC to informational conformity tendency. We found that participants receiving sham stimulation demonstrated the tendency to be consistent with the majority, and they were more likely to follow social information under congruent conditions than under incongruent conditions. Based on the findings of the sham group, we further assessed the stimulation effect. The results revealed that conformity tendency was significantly different in the three stimulations under incongruent conditions. The conformity tendency in cathodal stimulation was significantly higher than that in sham stimulation and anodal stimulation.

In the experiment, several factors contributed to the subjects’ conformity tendencies. First, at the behavioral level, as the absolute value of NPI increases, that is, as the absolute value of the number of people who make the same/different decision with the participants increases, it becomes easier for the participants to maintain/change their minds, which is in line with our intuition. Second, we found that participants tend to underestimate the correctness of their independent judgments. The participants’ estimation of their own correct rate is usually considered to directly reflects their subjective confidence in their own picture recognition ability (Moran et al., 2015; De Martino et al., 2017). In addition, more than one-third of the participants in the cathodal and sham groups thought that they did not perform as well as others, and few people in the anodal group thought the same way. From this, we may speculate that an individual’s subjective confidence level may be a reason for the different conformity tendencies, which is consistent with the findings of several recent studies (Mahmoodi et al., 2019; Wijenayake et al., 2020). Finally, we found that participants made more decisions consistent with the majority when they were informed of the right majority responses than the wrong majority responses, even though the participants did not actually know the correctness of the majority responses. Therefore, the decision-making behavior of the subjects after obtaining social information is probably not automatically implemented according to a certain “conformity rule” but adjusted according to specific contexts.

Based on the analysis of the Bayesian model, we found that when faced with a majority of different opinions (NPI = −3), subjects tend to rely on private information under anodal stimulation and tend to rely on social information under cathodal stimulation. In addition, compared to the sham and cathodal stimulation groups, the subjects in the anodal stimulation group were more likely to expect that their performance was better than others. Previous neuroscience studies found that subjective confidence plays an important role during decision-making in perceptual tasks and has a great correlation with the activity of the vmPFC (De Martino et al., 2017; Mahmoodi et al., 2018). Therefore, our findings are consistent with studies that suggest that people’s information integration is affected by subjective confidence (Moran et al., 2015; Boldt et al., 2019). Moreover, the RT analysis results also support this speculation. A majority of the same opinion increases subjective confidence, while a majority of different opinions can have the opposite effect (Hertwig, 2012; Koriat, 2012). Analysis of RT showed that the subjects’ reaction under congruent conditions was significantly faster than the reaction under incongruent conditions. This is also consistent with the research findings that there is a positive correlation between people’s decision-making time and their confidence level (Lebreton et al., 2015; Lopez-Persem et al., 2020). In summary, we suggest that confidence is likely to play an important mediating role in social conformity behavior. In particular, the anodal stimulus to the vmPFC enhanced the participants’ confidence and thus reduced the tendency to conform, while the cathodal stimulus increased the tendency of conformity by reducing the confidence level of the participants.

It should be noted that due to the particularity of the decision-making environment (social context), social conformity behavior may be driven by complex mechanisms rather than a unique reason (such as confidence). If informational conformity tendency can be fully explained by changes in confidence, then the effect of external influences on individual decision-making should be similar in both social and non-social environments. If the individual’s social conformity tendency is affected by factors other than confidence (such as priming effects, peer pressure, or reciprocity), then the conformity behavior in the social and non-social environments is likely to be different in the same environment. There should be a more diverse mechanism behind conformity. At present, some studies have supported the latter speculation by conducting comparative experiments on whether the opponent is a computer or a human (Germar et al., 2014; Mahmoodi et al., 2018). Unfortunately, our experiments based on this article cannot solve this problem. In the future, we can further explore the specific mediating role of confidence in social conformity through comparative research on social and non-social contexts.

Previous studies have used various experiments to elucidate the basic neurocognitive mechanism underlying conformity. Conformity was demonstrated not only using objective, perceptual tasks such as the one employed by Asch (1956) but also when subjective evaluative or preferential judgments were made (Berns et al., 2010; Izuma and Adolphs, 2013). These studies found that a large number of brain regions can be monitored during conformity, and the range of active brain regions is greatly affected by experimental formats. For example, the literature on conformity neural mechanisms based on visual tasks is often associated with functional changes in an occipital-parietal network (Berns et al., 2005), while the literature based on memory tasks is often found to be related to the activity in the ACC (Edelson et al., 2011; Deuker et al., 2013). Our results revealed that stimulation of the vmPFC did not change the subject’s ability to identify the dominant color. Thus, different conformity tendencies under three stimulations are more likely to be due to the subjects’ different weight distributions between the private and social information than to the subjects’ different abilities in the perceptual task.

Our findings showed that cathodal stimulation over the vmPFC significantly increased conformity tendency and decreased RT. Previous studies proposed that social conformity is related to some deep brain areas such as the insula and amygdala (Berns et al., 2005; Schnuerch and Gibbons, 2014). Cohen (2005) argues that the more recently evolved areas of the brain (including the prefrontal cortex) have developed to interact effectively with older structures. Meanwhile, functional connectivity has been observed between the vmPFC and the insula (Sutherland et al., 2013; Kirk et al., 2014; Moeller and Goldstein, 2014). Shamaytsoory et al. (2019) suggest that social alignment is mediated by a three-component feedback loop – an error-monitoring system that reacts to misalignment, an alignment system, and a reward system (including the vmPFC and ventral striatum) that is activated when alignment is achieved. Therefore, individuals under anodal and sham stimulation had a lower conformity tendency level and a longer RT, probably signifying that the tendency of conformity may depend on the interactions between deep brain regions and the cerebral cortex. Specifically, the cerebral cortex (such as vmPFC) is likely to play inhibitory and regulatory roles.

There are several limitations in our research. First, we followed some neuroscience research on the vmPFC and chose Fpz as the stimulation position. However, due to the close proximity of the vmPFC to the dmPFC and the ACC, the stimulation of Fpz may cause cortical excitability changes in other areas of the mPFC besides the vmPFC (Sellaro et al., 2015; Adenzato et al., 2019). Future studies may focus on refining and distinguishing the role of the mPFC in conformity behavior. Second, as discussed previously, we found that modulating the excitability of the vmPFC changed an individual’s conformity tendencies and resulted in some analyses of the possible moderating factors and neural circuits of the conformity, but these speculations cannot be demonstrated by a single experiment. In future research, we hope to further analyze the inner mechanism of this long-standing and common human behavior.

## Data Availability Statement

The datasets generated for this study are available on request to the corresponding author.

## Ethics Statement

The study conformed to the Declaration of Helsinki. All procedures performed in this study were reviewed and approved by the Ethics Committee of Zhejiang University of Finance and Economics. All participants provided their written informed consent to participate in this study.

## Author Contributions

YL, JW, HY, and JL designed the methodology and performed the experiments. YL and JL analyzed the data and wrote the manuscript. YL drew the figures. All authors contributed to the article and approved the submitted version.

## Conflict of Interest

The authors declare that the research was conducted in the absence of any commercial or financial relationships that could be construed as a potential conflict of interest.
